# Isolation and characterization of microparticles in sputum from cystic fibrosis patients

**DOI:** 10.1186/1465-9921-11-94

**Published:** 2010-07-09

**Authors:** Chiara Porro, Silvia Lepore, Teresa Trotta, Stefano Castellani, Luigi Ratclif, Anna Battaglino, Sante Di Gioia, Maria C Martínez, Massimo Conese, Angela B Maffione

**Affiliations:** 1 Department of Biomedical Sciences, University of Foggia, Via L.Pinto 1, Foggia, 71100, Italy; 2 Centro Regionale di Supporto FC, Ospedale "G. Tatarella", Via Trinitapoli, Cerignola, 71042, Italy; 3 INSERM U694, Université d'Angers, Rue Haute de Reculée, Angers, 49045, France

## Abstract

**Background:**

Microparticles (MPs) are membrane vesicles released during cell activation and apoptosis. MPs have different biological effects depending on the cell from they originate. Cystic fibrosis (CF) lung disease is characterized by massive neutrophil granulocyte influx in the airways, their activation and eventually apoptosis. We investigated on the presence and phenotype of MPs in the sputum, a rich non-invasive source of inflammation biomarkers, of acute and stable CF adult patients.

**Methods:**

Spontaneous sputum, obtained from 21 CF patients (10 acute and 11 stable) and 7 patients with primary ciliary dyskinesia (PCD), was liquefied with Sputasol. MPs were counted, visualized by electron microscopy, and identified in the supernatants of treated sputum by cytofluorimetry and immunolabelling for leukocyte (CD11a), granulocyte (CD66b), and monocyte-macrophage (CD11b) antigens.

**Results:**

Electron microscopy revealed that sputum MPs were in the 100-500 nm range and did not contain bacteria, confirming microbiological tests. CF sputa contained higher number of MPs in comparison with PCD sputa. Levels of CD11a^+^-and CD66b^+^-, but not CD11b^+^-MPs were significantly higher in CF than in PCD, without differences between acute and stable patients.

**Conclusions:**

In summary, MPs are detectable in sputa obtained from CF patients and are predominantly of granulocyte origin. This novel isolation method for MPs from sputum opens a new opportunity for the study of lung pathology in CF.

## Background

In cystic fibrosis (CF), the lung disease is characterized by high concentrations of neutrophil chemokines, such as IL-8, and a sustained accumulation of neutrophils in the airways [1,2], in presence and absence of detectable infection [3]. In CF airways, neutrophils undergo conventional activation and functional reprogramming [4-7]. For example, they show oxidative burst increase, enhanced production of leukotriens and elastase, increased IL-8 and decreased IL-1 receptor antagonist release (reviewed in [8,9]). However, the neutrophil response is not capable to clear bacteria from the CF airways ensuing in exaggerated apoptosis of neutrophils [10-13]. Furthermore, neutrophils are targeted by *Pseudomonas aeruginosa*, the main pulmonary pathogen associated with the disease. Neutrophils killed by the bacteria release proteases that disable any neighbouring viable neutrophils [14]. Thereafter, bacterial persistence and the products of the damaged neutrophils spur further neutrophil recruitment, inducing inflammation, tissue damage and then generation of an environment that allows continued infection.

Sputum is recognized as a very useful sampling method in CF for both research and clinical use aiding both the diagnosis and monitoring of lung disease inflammatory status. A great advantage of the technique is that it enables sampling of the airways in a non-invasive manner, in contrast with other methods such as bronchial biopsy, bronchial brushing and broncho-alveolar lavage, all of which require bronchoscopy, discomfort and risk that it entails [15]. Furthermore, sputum may contain protein/peptide components that could act as biomarkers of disease or its severity [16].

Microparticles (MPs) are small plasma membrane vesicles that are less than 1 μm released by several cell types (macrophages, platelets, endothelial cells, granulocytes, monocytes, lymphocytes) following chemical (cytokines, thrombin and endotoxin), physical (shear stress and hypoxia) [17] and apoptotic [18] stimuli. One of the first described roles for MPs was in the initiation and amplification of the coagulation cascade and furthermore they play a pivotal role in thrombosis, in the propagation of inflammation, modulation of vascular tone, angiogenesis, stem cell engraftment, and tumour metastasis. These MPs' effects depend on molecules harboured at their surface or within their cytoplasm due to their origin cell [18]. MPs are normally present in blood from healthy individuals but they increase in patients under pathological states associated with inflammation, such as sepsis [19], preeclampsia [20], metabolic syndrome [21], pulmonary arterial hypertension [22], and malaria [23], strengthening the notion that MPs may play a role in these diseases. The phenotype of circulating MPs is also different in different pathological states, and detection of its cellular origin may serve as a predictor or marker of the diseases [24].

Mutschler and colleagues, for the first time ever, showed the presence of MPs, derived from platelet, in pulmonary air-liquid interfaces in sedated pigs [25]. Recent investigation conducted in broncho-alveolar liquid fluid (BALF) has provided the characterization of intra-alveolar procoagulant MPs in patients with acute respiratory distress syndrome (ARDS) and hydrostatic pulmonary oedema. Intra-alveolar MPs from ARDS patients contain high levels of tissue factor, show an highly procoagulant activity, and are likely contribute to intra-alveolar fibrin formation, a critical pathogenic feature of acute lung injury [26]. To the best of our knowledge, no studies have been conducted to elucidate about the presence and role of MPs in other lung diseases. Since cellular activation and apoptosis, the main sources of MPs, are features of neutrophils in the CF airways, we have undertaken a study for the identification and characterization of MPs in the sputa of CF patients.

## Patients and Methods

### Study patients

The study was approved by, and performed in accordance with, the ethical standards of our institutional review boards on human experimentation. Written informed consent was obtained from each subject.

We enrolled 10 CF patients who consecutively had been admitted at the CF Center of the Hospital of Cerignola "G. Tatarella" for parenteral (i.v.) antibiotic therapy during acute respiratory exacerbation, and 11 stable CF patients. Exacerbation was defined as a deterioration in symptoms perceived by the patient and included an increase in cough, sputum production, dyspnoea, decline in forced expiratory volume in 1 sec (FEV_1_) compared with previous best, weight loss and fever [27]. Each patient was given a clinical score obtained from the sum of the individual parameters (0 = no symptom; 1 = moderate; 2 = severe). Serum C-reactive protein (CRP) was assessed as a marker of active inflammation [28]. CF patients were compared with 7 primary ciliary dyskinesia (PCD) patients.

Bacterial species in sputum specimens were identified accordingly to the North-American guidelines [29]. Sputum samples were directly spread-out in selective media, such as MacConkey agar for *Pseudomonas aeruginosa *and *Alcaligenes xilosoxidans*, manitol salt agar for *Staphylococcus aureus*, and BCSA for *Burkholderia cepacia *complex, and incubated at + 36 ± 1°C for a period of 18-72 h. Colonies were quantified and identified by classical (manual) phenotypical tests.

### MP isolation

Spontaneous sputum was collected in sterile cup and immediately processed. The sputum was washed with NaCl 150 mM, mixed with an equal volume (1:1) of Sputasol^® ^(SR 0233A, Oxoid Ltd, Hampshire, UK), and then incubated in a water bath at + 37°C for 15 min until visible homogeneous.

Processed sputum was centrifuged at 37 × *g *for 3 min the supernatant was centrifuged at 253 × *g *for 10 min and then recentrifuged at 253 × *g *for 20 min to remove the cells and large debris, respectively. Two hundred μl of each MP-containing supernatant were frozen and stored at-80°C until characterization by flow cytometry and microbiological tests.

Remaining MP-containing supernatant was centrifuged at 14,000 × *g *for 45 min to pellet MPs. MP pellet was subjected at two series of centrifugations at 14,000 × *g *for 45 min. Finally, MP pellet was replaced in 500 μl of 0.9% saline salt solution and stored at + 4°C until total counting.

### Characterization of MPs

MPs population was characterized in sputum supernatant, according to the expression of membrane-specific antigens. Anti-human CD11a labelling was used to numerate leukocyte MPs, while numeration of granulocyte MPs and monocyte/macrophage MPs was performed using anti-human CD66b and anti-human CD11b, respectively. Human IgM was used as isotype-matched negative control for CD66b staining, while IgG was used as isotype-matched negative control for CD11a and CD11b.

For these studies, 10 μl of supernatant MPs were incubated with 10 μl of specific antibody (1 μg/ml; FITC-conjugated; BioLegend, San Diego, CA). After 15 min of incubation at + 4°C, samples were diluted in 500 μl of 0.9% saline salt solution. Then, 10 μl of Flowcount beads were added to each sample and analyzed in a flow cytometer (Beckman Coulter coulter epics XL-MCL). Sample analysis was stopped after the count of 10,000 events.

### Bacteriological analysis

To rule out whether sputum supernatant staining was due to bacterial cells, supernatants, used for phenotypic characterization, were plated onto agar plates and kept at + 37°C for 16 hours.

As a further control, we evaluated two bacterial strains for cross-reaction with antibodies. *Pseudomonas aeruginosa *PAO1 strain [30] and *Staphylococcus aureus *ATTC strain 29213 were thawed and bacteria were recovered on agar-blood plates. One colony of *P. aeruginosa *and *S. aureus *were allowed to grow in 1 ml of Trypticase Soy Broth (TSB) (Difco, Becton Dickinson, Sparks, MD) or BBL™ brain heart infusion (BD Diagnostic Systems, Sparks, MD) respectively, for 1 hour at + 37°C. Bacteria were then incubated with 400 μg/ml gentamicin for 2 hours at + 37°C, and subsequently with anti-granulocyte antibodies under the same conditions of MPs, then finally analyzed by flow cytometry with the same settings used for MPs.

### Transmission electron microscopy

MPs contained in the supernatant of a processed CF sputum were subjected to a single centrifugation at 14,000 × *g *for 45 min. MP pellet was fixed in 4% glutaraldehyde in 0.1 M cacodylate buffer (pH 7.4) for 24 hours. The sample was then dehydrated in solutions of ethanol of increasing strength from 50%, 70%, 95% and 100% for 10 minutes in each solution. The sample was finally dehydrated in propylene oxide for 15 minutes. Finally, the sample was embedded in epoxy resin (Epon 12). After overnight polymerization, ultrathin sections (70 nm) were cut and examined in a JEOL (Tokyo, Japan) transmission electron microscope.

### Statistical analysis

Data are shown as mean ± SEM (Table 1) or medians (quantification and phenotype of MPs present in CF and PCD sputum). Statistical significance of differences between acute and stable groups of CF patients was evaluated by a two-tailed unpaired Student's *t*-test. To compare the number of MPs and the amount of different antigens the non-parametrical Mann-Whitney test was used. All data were analyzed using Prism 4 (GraphPad Software, Inc., La Jolla, CA). p values of less than 0.05 were considered significant.

**Table 1:** Baseline characteristics of patients.

	**Stable CF**	**Acute CF**	**p**	**PCD**
	
*N*	11	10		7
Age (years)	23.4 ± 4.3	24.5 ± 3.7		22.7 ± 3.5
Sex ratio (male:female)	7:4	2:8		3:4
F508del homozygous	5	2		/
F508del heterozygous	4	7		/
Other mutations	1	1		/
FEV_1 _(%)	57.8 ± 7.0	49.6 ± 6.4	0.53	75.2 ± 8.4
ΔFEV_1_	9.08 ± 2.5	0.07 ± 0.04	0.001	/
CRP (mg/dl)	0.53 ± 0.16	8.36 ± 2.87	0.01	/
Clinical score	7.3 ± 0.37	2.45 ± 0.31	0.001	/
Source of infection:				
*Pseudomonas aeruginosa*	11	6		3
*Staphylococcus aureus*	3	3		0
*Burkholderia cepacia complex*	0	2		0
*Alcaligenes xilosoxidans*	0	1		0

FEV_1_, forced expiratory volume in 1 second as % predicted. In 1 stable CF patient analysis of CFTR mutations is missing. Statistically significant differences between stable and acute CF patients are shown.

## Results

### Study patients

Characteristics of patient are summarized in Table 1. CF patients had more expiratory airflow obstruction, as measured by the FEV_1_% predicted, although not significantly different from PCD patients, and were more likely to be colonized with *Pseudomonas aeruginosa *and *Staphylococcus aureus*. In some CF patients, more than one bacterial strain colonized the same patient. The acute and stable groups of CF patients were well differentiated on the basis of decrease in FEV_1 _as compared with the best one in the last year (ΔFEV_1_), serum CRP, and clinical score (Table 1).

### Detection of MPs in CF sputa

**Figure 1:**
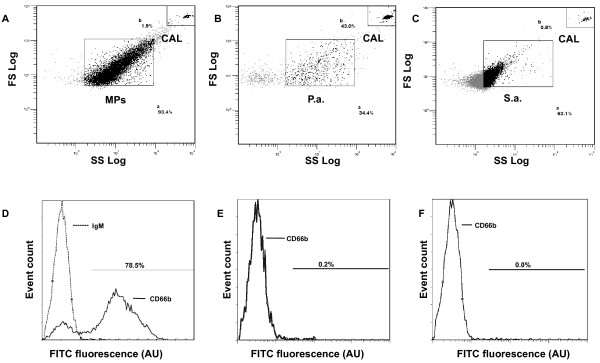
**Identification of MPs by flow cytometry**. Representative dot plots and histograms of MPs from sputum from CF patients and *P. aeruginosa *and *S. aureus*. MPs (**A**), *P. aeruginosa *PAO1 (**B**), *S. aureus *(**C**) and calibrator beads (10-μm; Beckman Coulter) are represented on a forward-scatter/side-scatter dot-plot histogram. MPs, defined as events with size of 0.1 to 1 μm in diameter, are gated in (a) window when compared with calibrator beads (CAL), gated in (b). Histograms showing the CD66b-FITC labelling of MPs from CF sputum (**D**), and the lack of staining obtained with control IgM, with *P. aeruginosa *PAO1 (**E**) or *S. aureus *(**F**).

Sputa liquefied with Sputasol, a dithiothreitol formulation, were centrifuged at low speed to remove large debris, and studied by flow cytometry analysis. MPs were readily identified in dot plots (Figure 1A) and were positive for CD66b antigen (Figure 1D). To discriminate whether bacteria or bacterial bodies could give such image, supernatants were plated onto agar plates and no bacterial growth was observed in sputa obtained from CF patients. However, to evaluate if bacteria could be stained by anti-granulocyte antibodies, *Pseudomonas aeruginosa *PAO1 were grown for 1 hour and then killed by gentamicin treatment. Although detectable in the same region of MPs (Figure 1B,), killed bacteria, analyzed by flow cytometry after antibody binding did not show any positivity for the antibody directed against CD66b (Figure 1E).

Also, *Staphylococcus aureus *ATTC strain 29213 was incubated with anti-granulocyte CD66b antibody. *S. aureus *was partially detectable in the same region of MPs (Figure 1C) but like *P. aeruginosa *did not show any positivity for the antibody directed against CD66b (Figure 1F). Therefore, we conclude that the staining of sputum supernatant was given only by MPs.

### Electron microscopy of sputum MPs

**Figure 2:**
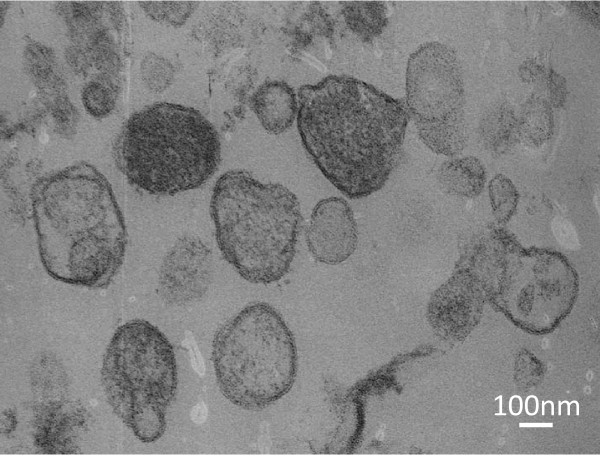
**Transmission electron microscopy of MPs from the sputum of a CF patient**. Multiple spherical particles, ranging from 100 to 500 nm, are visualized.

Figure 2 shows electron microscope picture of sputum-MPs from CF patients. Multiple spherical particles ranging in diameter from 100 to 500 nm were detected. Of note, no bacterial bodies were found associated with MPs.

### Levels of MPs in CF and PCD sputa

**Figure 3:**
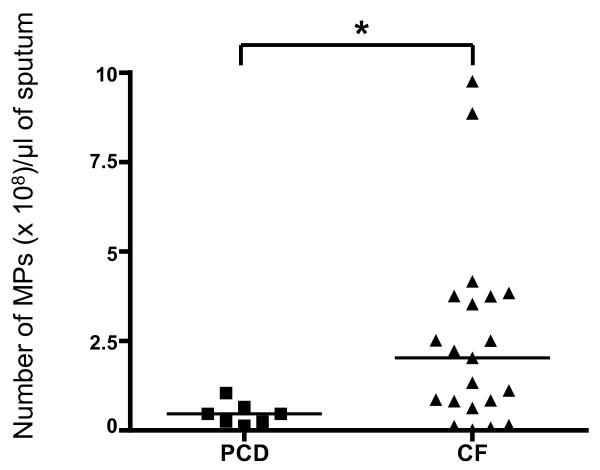
**Quantification of MPs in CF and PCD sputum**. Total MPs present in sputum of CF and PCD patients. CF patients (*n *= 21) show a significant higher number of MPs respect to PCD patients (*n *= 7). *p = 0.00297.

We evaluated the level of MPs in the sputum of 21 CF patients compared with the sputum of 7 PCD patients. Although heterogeneous, comprising both stable and acute patients, the CF group showed a significantly higher number of MPs than the PCD group (Figure 3).

### MP phenotype

**Figure 4:**
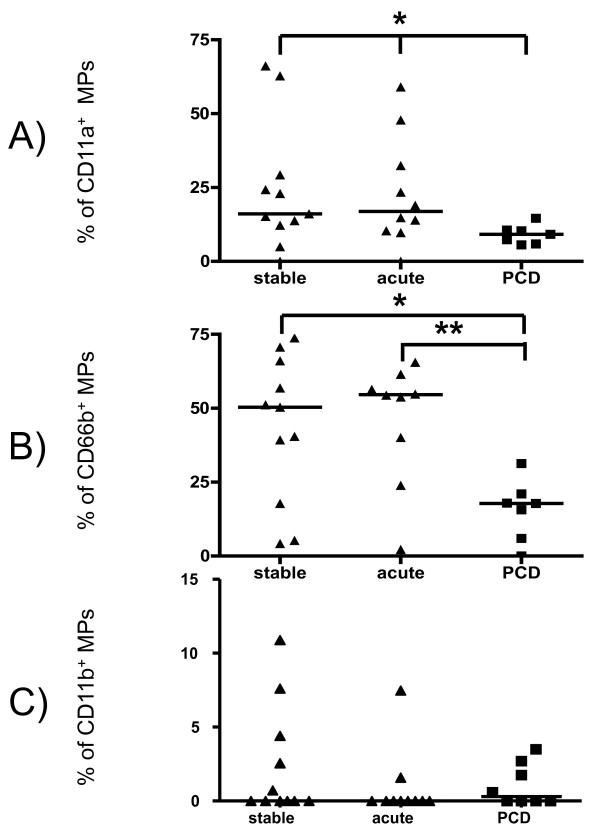
**Phenotype of MPs present in sputa of acute and stable CF and PCD patients**. In acute and stable CF patients, the number of MPs staining positive for FITC-conjugated antibodies directed against CD11a are significantly higher than in PCD patients (**A**). Stable CF vs. PCD: *p = 0.0464. Acute CF vs. PCD: *p = 0.0431. MPs positive to CD66b, both in acute and in stable phase of CF, are significantly elevated in respect to PCD (**B**). Stable CF vs. PCD: *p = 0.0373. Acute CF vs. PCD: **p = 0.0046. Positivity of MPs to CD11b is not significantly different in the three groups of patients (**C**).

MP phenotype was analyzed by evaluating the presence of antigens representing different cell types: CD11a for leukocytes, CD66b for granulocytes, CD11b for monocyte/macrophages. In CF patients, amount of MPs expressing CD66b (median value of 53.8%) was higher than those expressing CD11a (median value of 16.1%) and CD11b (median value of 0%). Comparison of all CF patients versus PCD patients showed that amounts of MPs expressing CD66b and CD11a were significantly higher in CF than in PCD (CD66b: p = 0.0068; CD11a: p = 0.0226) (data not shown). No differences in the three populations of MPs were found between patients in acute and stable phase of CF. However, both acute and stable patients showed significantly higher levels of MPs expressing CD66b and CD11a in comparison to PCD patients (for CD66b: stable CF vs. PCD: p = 0.0373; acute CF vs. PCD: p = 0.0046. For CD11a: stable CF vs. PCD: p = 0.0464; acute CF vs. PCD: p = 0.0431; Figure 4).

## Discussion

Simple and non-invasive biomarkers of lung inflammation in CF are needed to monitor disease progression, identify exacerbations, and evaluate the efficacy of novel therapies [31]. Sputum is a rich, non-invasive source of biomarkers of inflammation and infection, and has been used extensively to assess inflammation in the CF airways (reviewed in [15]). There is compelling evidence from small single-centre studies supporting an association between sputum biomarkers and disease status in CF. Recently, a multicenter cross-sectional study has found significant negative correlations between FEV_1_% predicted and spontaneously expectorated sputum inflammatory markers including free elastase, IL-8, neutrophil counts, and percent neutrophils [32].

In this study we provide evidence, for the first time, of the presence of MPs in sputa obtained from CF patients. The membrane composition of MPs reflects the plasma membrane of the original cell at the precise moment of MPs production and thus allows the characterization of the cellular source [33] using antibodies directed against these specific epitopes. Our data strongly support the notion that MPs are derived from granulocytes, while the presence of MPs derived from monocyte-macrophages is negligible. Although there are several potential sources of sputum MPs, including erythrocytes, platelets, and epithelial cells, our data suggest that granulocytes are the predominant source of MPs in CF. Our findings are consistent with massive influx of neutrophils into CF airways and their accumulation on the surface of the airway epithelium [34]. In this environment, neutrophils are activated by bacterial products, pro-inflammatory cytokines, and chemokines. Neutrophils undergo apoptosis, as normally happens in acute inflammation, but also post-apoptotic necrosis, releasing toxic enzymes and oxygen radicals. MPs in CF sputa likely reflect both activation and apoptosis of neutrophils. In order to evaluate the effect of bacterial infection on MPs production, it would be interesting to compare subgroups based on the presence or absence of infection with *Pseudomonas aeruginosa *or other bacterial strains, therefore further studies with larger number of patients are needed. PCD patients were selected as controls because healthy donors do not produce spontaneous sputum; moreover, PCD patients have similar respiratory infections to CF patients. In PCD, neutrophilic lung inflammation, incidence of lung infection, offending organisms, development of bronchiectasis and longitudinal declines in lung function are similar to CF but appear to be delayed, and serious lung disease tends to develop later in life [35-37]. This could be the reason for a significantly less presence of granulocyte-derived MPs in PCD when compared with CF patients.

The mechanisms of MP formation are complex and not completely elucidated. Following cell activation or apoptosis, MPs formation is dependent on a sustained rise in the cytosolic Ca^2+ ^concentration with the consequent activation of different cytosolic enzyme relevant to MPs formation. Calpain is one of the most important enzyme and has several actions in MPs generation including cleaving of cytoskeletal filaments, facilitating microparticle shedding, and activating apoptosis through procaspase-3 [38]. These changes result in cytoskeletal reorganization, loss of the asymmetric distribution of aminophospholipid, membrane blebbing and MP formation [39].

Some release of shedding vesicles takes place from resting cells, however the rate of the process increases dramatically upon stimulation [40]. Ca^2+ ^is not the only second messenger involved. In various cell types, in fact, phorbol ester activation of protein kinase C (PKC) is also effective. In PC12 cells, shedding vesicles are released upon application of phorbol esters and not of Ca^2+ ^ionophores. The purinergic receptors of ATP, a ligand released by many cell types, have an important role. In dendritic cells, macrophages and microglia, activation of the purinergic receptor channel, P2X7, was found to induce intense release of MPs. In other cell types (such as PC12 and platelets), activation of the P2Y receptors coupled with the Gq protein was found to be effective.

Electron or confocal laser scan microscopy can be used for better characterization of morphological features or visualization of MPs. Indeed, here we show that sputum MPs display a range between 100 and 500 nm, larger than that previously shown for MPs obtained from edema fluid of a patient with ARDS [26]. However the most widely used method for studying MPs is flow cytometry due to its simplicity and the wealth of information that can be gleaned from the population under study [17]. The major advantage of flow cytometry is staining of MPs to determine the origin/cellular source of MPs. In addition, flow cytometry can also be used to enumerate blood MPs by adding a known number of fuorescent or non fuorescent latex particles to the sample prior to performing analysis [41].

Microvesicles also originate from the endosomal membrane compartment after fusion of secretory granules with the plasma membrane, where they exist as intraluminal membrane-bound vesicles called exosomes. These exosomes are released from cells during exocytosis of secretory granules together with the proteins present inside these granules. MPs are released from the surface of membrane during membrane blebbing in a calcium flux and calpain-dependent manner and are relatively large (100 nm-1 μm). In contrast smaller exosomes that are more homogeneous in size (30-100 nm) are released from the endosomal compartment [42]. In the only paper reporting a direct comparison, the shedding vesicles of platelets 'could be detected by flow cytometry but not the exosomes, probably because of the smaller size of the latter' [43].

MPs contain numerous proteins and lipids similar to those present in the cell membranes from which they originate. Furthermore, as MPs' membranes engulf some cytoplasm during membrane blebbing, they may also contain proteins derived from it, mRNA, and, as recently demonstrated, microRNA (miRNA) [44]. It is now emerging that miRNAs may play a key role in host defence and inflammation [45,46]. Moreover, MPs may "hijack" infectious particles (e.g. human immuno deficiency virus (HIV) or prions) from the cytoplasm or possibly even whole intact organelles such as the mitochondria [42].

A question remains unresolved: whether MPs present in CF sputa have functional consequences on the pathophysiology of CF lung disease. Recent data bring the evidence that MPs can transfer message from different type of cells. The mechanisms by which MPs may influence biology of target cell could be different; MPs may (i) stimulate other cells by surface-expressed ligands acting as a signalling complex, (ii) transfer surface receptors from one cell to another, (iii) deliver proteins, mRNA, miRNAs, and bioactive lipids into target cells or (iv) serve as a vehicle for the transfer of infectious particles (e.g. HIV, prion) [42].

## Conclusions

Sputum through its inflammatory cell, bacterial, volatiles, mucin and protein content represent an important tool for the diagnosis and monitoring of CF and other respiratory diseases, beside for the study of disease pathogenesis and its treatment. Measurement of these components is largely sophisticated and quantifiable, and the search for novel biomarkers of CF airways disease in this biofluid is under way [16,47]. The finding of MPs in sputum raises some intriguing questions on the pathophysiological role of MPs in pulmonary epithelium. Our data strongly support the notion that MPs are derived from granulocytes of CF patients, and this correlates with massive influx of neutrophils into CF airways and their accumulation on the surface of the airway epithelium [34]. In this environment, neutrophil-derived MPs could contribute to self-perpetuating inflammatory cycle, and may account for the exaggerated proinflammatory response of cells in CF patients.

Independently of the potential role played by MPs in CF, taking in consideration the fact that CD66b^+ ^MPs are present in higher level in CF than in PCD sputum, they might be considered as biological markers of this pathology. Taken together, these data open a new opportunity for the study of lung pathology.

## Competing interests

The authors declare that they have no competing interests.

## Authors' contributions

CP performed all experimental steps and wrote the manuscript; SL, TT, SC and SDG provided experimental assistance; LR and AB enrolled patients; ABM, MC and MCM conceived the study, supervised this project and participated in its coordination and helped to draft the manuscript. All authors read and approved the final manuscript
